# Edge-Distilled and Local–Global Feature Selection Network for Hyperspectral Image Super-Resolution

**DOI:** 10.3390/s26031055

**Published:** 2026-02-06

**Authors:** Xinzhao Li, Mengzhe Fan, Xiaoqing Zheng, Jiandong Shang

**Affiliations:** 1 National Supercomputing Center in Zhengzhou, Zhengzhou University, Zhengzhou 450001, China; ielixinzhao@zzu.edu.cn (X.L.); mzfan@zzu.edu.cn (M.F.); zhengxq@zzu.edu.cn (X.Z.); 2 The School of Computer and Artificial Intelligence, Zhengzhou University, Zhengzhou 450001, China

**Keywords:** hyperspectral image, super-resolution, edge-distilled, local–global feature selection, deep learning

## Abstract

In recent years, the methods based on convolutional neural networks have achieved significant progress in hyperspectral image super-resolution. However, existing methods still face two key challenges: (1) they fail to fully extract edge detail information from hyperspectral images; (2) they struggle to simultaneously capture local and global features. To address these issues, we propose an Edge-Distilled and Local–Global Feature Selection network (EDLGFS) for hyperspectral image super-resolution. This network aims to effectively leverage edge details and local–global features, thereby enhancing super-resolution reconstruction quality. Firstly, we design an edge-guided super-resolution network based on knowledge distillation. This network transfers edge knowledge to improve the reconstruction. Secondly, we propose a Local–Global Feature Selection mechanism (LGFS), which integrates convolutions of different sizes with the self-attention mechanism. This design models spatial correlations across features with different receptive fields, achieving efficient feature selection to more effectively capture local and global features. Finally, we propose a dynamic loss mechanism to more effectively balance the contribution of each loss term. Extensive experimental results on three public datasets demonstrate that the proposed EDLGFS achieves superior super-resolution reconstruction quality.

## 1. Introduction

Hyperspectral images (HSIs) are typically acquired by capturing tens to hundreds of continuous spectral bands within near-infrared, mid-infrared, visible light, and other bands of the electromagnetic spectrum [1]. Unlike traditional RGB or multispectral images, HSIs possess extremely high spectral resolution, enabling them to capture detailed spectral characteristics of target objects at every spatial location, which allows for precise material identification. Therefore, HSIs find extensive applications in various fields, such as target detection [2,3], mineral exploration [4], and medical diagnostics [5]. Furthermore, core hyperspectral analysis tasks including spectral unmixing and fine-grained land cover classification also heavily rely on high-quality HSIs. Recent advanced methods, such as spatial-channel multiscale Transformer networks for unmixing [6] and multi-scale memory networks for detection [7], further demonstrate the growing demand for precise spatial–spectral representations. However, due to fundamental imaging system constraints, achieving high spectral resolution often compromises spatial resolution. This limits the performance of the aforementioned applications [8]. Therefore, super-resolution (SR) techniques are required to enhance the spatial resolution of HSIs without hardware upgrades, thereby providing a superior data foundation for advanced computational models.

Recently, hyperspectral image super-resolution (HSISR) has become a vibrant research topic. Wang et al. [9] provided a systematic review of HSISR. This review categorizes HSISR techniques into fusion-based techniques [10,11] and single-image SR techniques [12,13], and highlights key challenges including spectral distortion and edge preservation. Fusion-based techniques typically integrate low-resolution (LR) HSIs with complementary high-resolution (HR) images, such as RGB or multispectral images. This produces results with enhanced spatial details while preserving spectral fidelity. However, these methods depend on HR auxiliary images and require complete registration with the HSIs. These requirements pose significant challenges in practical applications. Single-image SR does not depend on auxiliary images and only utilizes the LR HSIs to improve spatial resolution, making it more flexible in practical applications.

In the past, traditional single HSISR methods primarily relied on manually defined prior constraints and assumptions, such as sparse regularization [14] and three-dimensional total variation [15]. These prior constraints often fail to capture the complex features of HSIs, thereby limiting the models’ generalization ability. Recent surveys, such as Wang et al. [9], have also noted that prior constraints are often too simplistic to model complex real-world scenes. This has motivated a shift toward data-driven deep learning approaches.

Currently, convolutional neural networks (CNNs) have been extensively applied to natural image SR [16,17]. The core principle lies in extracting complex structural features in images through multiple convolutional layers and feature learning mechanisms [18,19,20]. Due to the outstanding performance of CNNs in image SR, researchers have extended their application to HSISR. The existing deep learning-based HSISR networks are mainly divided into 2D CNNs [21,22,23] and 3D CNNs [24,25,26,27]. Two-dimensional CNNs conduct independent convolution operations on each spectral band, which ignores spectral continuity. Three-dimensional CNNs can explore both spatial context and spectral correlations between adjacent bands. However, 3D CNNs fail to capture long-range spatial correlations and spectral similarities. Additionally, 3D convolutions introduce significant computational complexity.

To overcome these limitations, the Transformer architecture has been introduced into HSISR in recent studies [28,29]. As a deep learning model based on self-attention mechanisms, the Transformer excels at capturing global information and long-range dependencies. In contrast, CNNs efficiently extract fine local features due to their local receptive fields. Their complementary strengths in feature extraction have been applied to HSISR. For example, SwinIR [30] and SST [31] incorporate convolutional layers after multiple Transformer modules, combining the local inductive bias of CNNs with the global attention capability of the Transformer. Based on these complementary features, the DSSTSR network [32] designs the dual self-attention Swin Transformer, which utilizes spatial–spectral self-attention to minimize spectral distortion while extracting spatial features.

However, these methods typically suffer from two main limitations. First, they fail to fully utilize fine edge details. Second, they cannot model both local and global features simultaneously. These issues not only degrade visual quality but also impair the performance of downstream vision tasks that rely on precise spatial and spectral information [33], such as target detection, land cover classification, and fine-grained material identification. Therefore, it is crucial to develop an SR method that can effectively preserve edges and captures both local and global features.

Inspired by this, we propose an Edge-Distilled and Local–Global Feature Selection network (EDLGFS) for HSISR. This network adopts a parallel dual-path architecture. The main branch captures the complex local–global features and the auxiliary edge branch focuses on extracting and refining edge details. This separation treats edge information as explicit prior knowledge. It prevents edge details from being suppressed by other features, which is a common issue in single-stream designs. A core component of the network is the intermediate supervision strategy. We design a dynamic loss mechanism between the two branches. This guides the main branch to learn the edge details from the auxiliary branch instead of directly fusing features. During the intermediate feature extraction, we propose a Local–Global Feature Selection (LGFS) module. It combines convolutions of different sizes with self-attention to model spatial correlations among features of different receptive fields. This module achieves efficient feature selection, thereby capturing local–global features more effectively. Extensive experiments on three public datasets demonstrate that EDLGFS achieves superior SR reconstruction quality.

The core innovation of this paper lies in the integrated design of the overall architecture. It incorporates edge knowledge distillation, local–global feature selection, and a dynamic loss mechanism. In this study, our main contributions are summarized as follows:

(1) We propose a super-resolution network using an edge distillation architecture. The auxiliary edge branch transfers knowledge only during training and is removed for inference. This guides the main branch to learn edge details without increasing computational complexity.
(2) We design a Local–Global Feature Selection (LGFS) module. This module combines convolutions of different sizes with the self-attention. This fully captures local–global features through efficient feature selection.
(3) We introduce a dynamic edge loss mechanism. By assigning learnable weights to different loss terms, it adaptively balances edge detail preservation and overall reconstruction. This method enhances training stability and improves the model’s reconstruction performance.

The structure of the remaining part of this article is as follows: Section 2 reviews existing SR methods for HSIs. Section 3 details the proposed EDLGFS method. Section 4 presents the datasets, experimental results and ablation studies. Finally, Section 5 concludes the paper.

## 2. Related Work

### 2.1. CNN-Based Single HSISR

Deep learning techniques have recently driven significant progress in single HSISR. Consequently, numerous convolutional neural networks (CNNs) have been developed for this task [12,21,23,24,34]. Li et al. [21] proposed an HSISR method combining a spatial constraint (SCT) strategy with a deep spectral difference CNN (SDCNN), which effectively enhances spatial resolution while preserving spectral integrity. Jia et al. [23] proposed a Spectral–Spatial Network (SSN) that divides the reconstruction task into a spatial section, enhanced by a maximum variance principle, and a spectral section optimized via a spectral angle error loss function to preserve spectral signatures. Yuan et al. [35] transferred knowledge from natural images to learn a low-to-high-resolution mapping for HSIs. They also used collaborative non-negative matrix factorization (CNMF) to preserve spectral characteristics. In order to capture the spectral continuity across adjacent bands in HSIs, Mei et al. [24] proposed a 3D full CNN (3D-FCNN) for HSISR. Li et al. [36] proposed a Mixed Convolution Network (MCNet) for HSISR. It combines 2D and 3D convolutions to better capture latent spatial features. Liu et al. [37] proposed a fully 3D U-Net (F3DUN) with skip connections for deep multi-scale feature extraction. Their work demonstrated the efficacy of pure 3D CNN for HSISR. Wang et al. [9] provided a comprehensive review of deep learning-based HSISR methods. They categorized techniques into single image, panchromatic image-assisted, and multispectral image-assisted approaches. Additionally, they summarized common datasets, metrics, and applications. Hu et al. [38] proposed a novel HSISR method named SNLSR, which recasts the SR task into the abundance domain. It utilizes a spatial-preserving decomposition network and spectral non-local attention to restore high-frequency details. Li et al. [39] proposed a Test-Time Training framework for HSISR that incorporates a novel self-training strategy and Spectral Mixup augmentation, effectively overcoming data scarcity to significantly enhance reconstruction performance across diverse re-al-world scenarios. However, these CNN-based methods primarily extract local features. They often fail to effectively model long-range spatial correlations and spectral similarities.

### 2.2. Transformer-Based Single HSISR

The Transformer possesses robust long-range dependency modeling capabilities and is widely applied in HSISR tasks. Liu et al. [40] proposed an innovative method to address HSISR by fusing a Transformer with 3D CNN. Their Interactformer model uses a dual-branch architecture. It effectively preserves spectral integrity while enhancing spatial details. Chen et al. [41] proposed a Multi-Scale Deformable Transformer (MSDformer). This method combines the local feature extraction strengths of CNNs with the global modeling capabilities of Transformers. It utilizes a Multi-Scale Spectral Attention Module to precisely extract local multi-scale features and employs a Deformation Convolution-based Transformation Module to effectively capture global long-range dependencies. Zhang et al. [42] proposed an efficient Transformer model named ESSAformer, which incorporates a linear complexity attention mechanism based on the spectral correlation coefficient (SCC). This approach not only reduces computational cost but also enhances reconstruction quality. Chen et al. [43] introduced a novel Cross-range Spatial–Spectral Transformer (CST). This method employs cross-attention mechanisms across spatial and spectral dimensions to capture long-range spatial–spectral dependencies. Zhang et al. [44] proposed a spatial–spectral aggregation Transformer that incorporates diffusion priors. It extracts prior features using a self-supervised diffusion model. By integrating an adaptive fusion module, it significantly improves reconstruction quality. However, these methods primarily focus on enhancing overall reconstruction quality. They often fail to fully exploit fine features like image edges.

### 2.3. Edge-Guided Single Image SR

Researchers have explored various edge-guided strategies to fully exploit edge details in HSIs. For example, Yang et al. [45] proposed a deep edge-guided recurrent residual network named DEGREE, which progressively restores high-frequency details using recurrent residuals and edge information. Zhao et al. [46] proposed G-RDN, which enhances image reconstruction quality by utilizing spatial gradients to highlight edges and textural details. Wang et al. [47] introduced the Edge-Guided Super-Resolution Network (EGSRN). This network employs an Edge Net module to explicitly extract edge features from LR images. It then integrates edge and image features through multi-layer Feature Extraction Modules and an Edge Information Fusion mechanism. However, these methods often fail to effectively capture local–global features, limiting the completeness of feature representation. This paper proposes an Edge-Distilled and Local–Global Feature Selection network (EDLGFS) to address these challenges. This network efficiently extracts fine edge features while simultaneously capturing local features and global contextual information.

## 3. Materials and Methods

### 3.1. Overall Network

**Figure 1:**
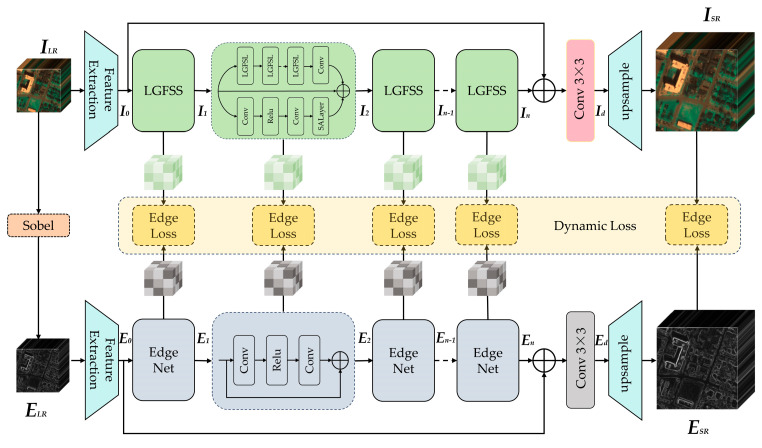
The overall architecture of EDLGFS.

Figure 1 depicts the overall framework of EDLGFS, which consists of two parallel branches. The main branch learns complex local–global features, while the auxiliary edge branch focuses on extracting and refining edge details. The auxiliary edge branch guides the main branch through knowledge distillation. We denote the input LR HSIs as $I_{LR}\in R^{C\times H\times W}$, the original HR HSIs as $I_{HR}\in R^{C\times sH\times sW}$, and the reconstructed HSIs as $I_{SR}\in R^{C\times sH\times sW}$, where H and W represent height and width respectively. s denotes the SR scaling factor, and C denotes the number of spectra bands. We first extract edge maps from $I_{LR}$ for each spectral band using the Sobel operator. This is expressed as follows:

(1) $$E_{LR}=h_{sobel}\left(I_{LR}\right)$$

where $h_{sobel}(\cdot )$ represents the Sobel edge extraction function. $E_{LR}\in R^{C\times H\times W}$ represents the edge image extracted from $I_{LR}$. The shallow features are extracted through a 3 × 3 convolution layer. This process can be represented as follows:

(2) $$I_{0}=h_{c}\left(I_{LR}\right)$$

(3) $$E_{0}=e_{c}(E_{LR})$$

where $h_{c}(\cdot )$ and $e_{c}(\cdot )$ denote shallow feature extraction functions and $I_{0}$ and $E_{0}$ represent the corresponding shallow features. Subsequently, $I_{0}$ is processed through a series of Local–Global Feature Selection Stages (LGFSSs) to extract deep features. Meanwhile, $E_{0}$ is processed by a sequence of Edge Net modules to extract deep edge features.

The LGFSS comprises two parallel branches. The first branch employs a series of Local–Global Feature Selection layers (LGFSLs) followed by a 3 × 3 convolution. The second branch consists of two consecutive 3 × 3 convolutional layers and a spectral attention layer [43]. The features from both branches are adaptively fused via residual connections. The deep feature $I_{n}$ can be expressed as follows:

(4) $$I_{n}=h_{n}(I_{n-1}),n=1,2,\ldots ,N$$

where $h_{n}(\cdot )$ represents the function of the *n*-th LGFSS, and $I_{n}$ represents the corresponding deep features extracted by the *n*-th LGFSS. In parallel, $E_{0}$ is processed by an equal number of Edge Net modules to extract hierarchical edge features. Each Edge Net module consists of two consecutive 3 × 3 convolutional layers and residual connections. Deep edge feature extraction is expressed as follows:

(5) $$E_{n}=e_{n}(E_{n-1}),n=1,2,\ldots ,N$$

where $e_{n}(\cdot )$ denotes the *n*-th Edge Net function, and $E_{n}$ represents the deep edge features extracted by the *n*-th Edge Net. We employ an edge loss function ($L_{f}^{n}(\Theta )$) to connect all levels of $I_{n}$ and $E_{n}$ separately, so that $I_{n}$ can learn the edge feature from $E_{n}$. Then, the outputs from the last layer ($I_{n}$ and $E_{n}$) are processed via skip connections and a convolutional layer. The final deep features are represented as follows:

(6) $$I_{d}=Conv(I_{n}+I_{0})$$

(7) $$E_{d}=Conv(E_{n}+E_{0})$$

where $I_{d}$ and $E_{d}$ represent the deep features. Finally, the image reconstruction layer processes the deep features to generate the SR image, which is represented as follows:

(8) $$I_{SR}=h_{up}\left(I_{d}\right)$$

(9) $$E_{SR}=e_{up}(E_{d})$$

where $h_{up}\left(\cdot \right)$ and $e_{up}(\cdot )$ denote the upsampling operations via the PixelShuffle method. $I_{SR}$ and $E_{SR}$ represent the reconstructed HSI and edge map, respectively. Finally, $I_{SR}$ learns image-level edge information from $E_{SR}$ through an edge loss function ($L_{ST}(\Theta )$).

### 3.2. Local–Global Feature Selection (LGFS)

**Figure 2:**
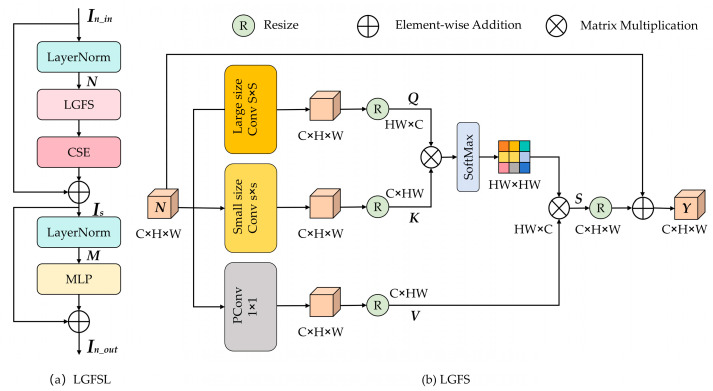
Structure of the designed LGFSL and LGFS.

To fully capture the local–global features, we introduce the Local–Global Feature Selection layer (LGFSL), inspired by the Metaformer architecture [48]. Additionally, we incorporate the Cross-Scope Spectral Self-Attention module (CSE) [43] within LGFSL to extract cross-range spectral correlations in HSIs. As shown in Figure 2a, LGFSL consists of two LayerNorm layers, a Local–Global Feature Selection (LGFS) module, a CSE module, and a Feed-Forward Network (MLP). These modules are connected through two residual structures.

For the input feature $I_{n\_in}\in R^{C\times H\times W}$, the whole process of LGFSL is represented as follows:

(10) $$N=LN(I_{n\_in})$$

(11) $$I_{s}=CSE(LGFSL(N))+I_{n\_in}$$

(12) $$M=LN(I_{s})$$

(13) $$I_{n\_out}=MLP(M)+I_{s}$$

where $I_{n\_out}\in R^{C\times H\times W}$ denotes the output feature, LN(·) denotes LayerNorm, LGFSL(·) denotes LGFSL, CSE(·) denotes CSE, and MLP(·) denotes the multi-layer perceptron module.

Due to the ability to capture long-range dependencies [49,50,51,52], self-attention mechanisms have been widely applied in many SR methods. However, these methods often fail to effectively capture local–global features. We propose the Local–Global Feature Selection (LGFS) module to address this issue. Its structure is shown in Figure 2b.

Given that $N\in R^{C\times H\times W}$ denotes the input feature of LGFS, then, N is projected into query ($Q\in R^{C\times H\times W}$), key ($K\in R^{C\times H\times W}$), and value ($V\in R^{C\times H\times W}$) through a large kernel convolution, a small kernel convolution, and a point-wise convolution, respectively. It can be expressed as follows:

(14) $$Q=W_{q}(N),K=W_{k}(N),V=W_{\nu }(N)$$

where $W_{q}(\cdot )$, $W_{k}(\cdot )$, and $W_{\nu }(\cdot )$ denote large kernel, small kernel, and point-wise convolutions, respectively. Large kernels have a larger receptive field, enabling them to capture broader contextual information and enhancing global modeling capabilities. Small kernels focus on local detailed features and refined spatial structures. By combining small and large kernels, the network can efficiently capture local–global features. Next, after transposing and flattening the spatial dimensions of Q, K, and V, they are reshaped into $Q\in R^{HW\times C}$, $K\in R^{C\times HW}$, and $V\in R^{C\times HW}$. Then, we obtain the attention score by matrix multiplication:

(15) attn_scores=Q⊗K

where $attn\_scores\in R^{HW\times HW}$ represents the similarity between all spatial positions, and the symbol ⊗ represents matrix multiplication. Then, we apply the softmax function to obtain the attention weights, which is represented as follows:

(16) $$attn\_weight=h_{Soft}\left(attn\_scores\right)$$

where $attn\_weight\in R^{HW\times HW}$ represents the attention weights. The softmax function is applied along the last dimension to ensure that the sum of weights at each position is 1. Then the feature V is weighted and aggregated using the attention weight, which is represented as follows:

(17) $$S=attn\_weight⊗V^{T}$$

Finally, $S\in R^{HW\times C}$ is transposed and reshaped into $S\in R^{C\times H\times W}$, which is added to the original input N to realize the residual connection. It is expressed as follows:

(18) Y=S+N

where $Y\in R^{C\times H\times W}$ represents the module output. The residual connection facilitates gradient flow and stabilizes the training process. The LGFS module enables effective local–global feature selection, thereby enhancing SR reconstruction quality.

### 3.3. Dynamic Loss Mechanism

At present, many works have demonstrated that $l_{1}$ and $l_{2}$ loss functions have achieved good results in SR tasks [53]. The $l_{2}$ loss function encourages finding a reasonable pixel-level average, which may lead to too smooth results. The $l_{1}$ loss function can better balance the error distribution. Therefore, we employ the $l_{1}$ loss function for both assessing the quality of the SR reconstruction and guiding the main network to learn edge features. Additionally, we designed learnable dynamic weights to more effectively balance the contribution of each loss term.

Given $I_{n}\in R^{C\times H\times W}$ represents the deep features obtained by the *n*-th LGFSS block of the main network, and $E_{n}\in R^{C\times H\times W}$ represents the deep edge features obtained by the *n*-th Edge Net block of the auxiliary edge network. Then, the edge loss $L_{f}^{n}(\Theta )$ of the *n*-th deep feature and the total edge loss $L_{f}(\Theta )$ of all deep features can be expressed as follows:

(19) $$L_{f}^{n}(\Theta )=\frac{1}{M}\sum_{m=1}^{M}\;{∥I_{n}^{m}-E_{n}^{m}∥}_{1}$$

(20) $$L_{f}(\Theta )=\sum_{n=1}^{N}\;L_{f}^{n}(\Theta )$$

where M denotes the batch size, N indicates the number of deep features in the network, $I_{n}^{m}$ denotes the *n*-th deep feature of the *m*-th image, $E_{n}^{m}$ represents the *n*-th deep edge feature of the *m*-th image, and Θ represents the learnable parameters in our network.

In addition, for the reconstructed HSIs $I_{SR}$ and the reconstructed edge image $E_{SR}$, we use the $l_{1}$ loss function to guide the network to learn the reconstructed edge features, which can be expressed as follows:

(21) $$L_{ST}(\Theta )=\frac{1}{M}\sum_{m=1}^{M}\;{∥I_{SR}^{m}-E_{SR}^{m}∥}_{1}$$

where M denotes the batch size and $I_{SR}^{m}$ and $E_{SR}^{m}$ represent the *m*-th reconstructed HSIs and the *m*-th reconstructed edge image respectively.

In addition to the above edge loss, we also designed loss functions $L_{SD}(\Theta )$ and $L_{TD}(\Theta )$ for data monitoring. $L_{SD}(\Theta )$ is to compare the reconstructed HSIs with the real HR HSIs, and $L_{TD}(\Theta )$ is to compare the reconstructed edge image with the real edge image. $L_{SD}(\Theta )$ and $L_{TD}(\Theta )$ can be expressed as follows:

(22) $$L_{SD}(\Theta )=\frac{1}{M}\sum_{m=1}^{M}\;{∥I_{SR}^{m}-I_{HR}^{m}∥}_{1}$$

(23) $$L_{TD}(\Theta )=\frac{1}{M}\sum_{m=1}^{M}\;{∥E_{SR}^{m}-E_{HR}^{m}∥}_{1}$$

where M is the number of inputs in the training batch, $I_{SR}^{m}$ and $I_{HR}^{m}$, respectively, represent the *m*-th reconstructed HSIs and real HR HSIs, $E_{SR}^{m}$ and $E_{HR}^{m}$ represent the *m*-th reconstructed edge image and real HR edge image, respectively, and $E_{HR}^{m}$ is extracted from $I_{HR}^{m}$ by using Sobel operator.

We define the total loss function for the network as follows:

(24) $$L_{total}(\Theta )=\lambda_{1}L_{SD}+\lambda_{2}(L_{f}+L_{ST}+L_{TD})$$

where $\lambda_{1}$ and $\lambda_{2}$ are the dynamic learnable weights we designed, aiming to balance the contribution of different loss terms.

## 4. Experiments and Results

### 4.1. Datasets

To evaluate our method, we conduct experiments on three public HSI datasets: Houston, Pavia Center, and Chikusei.

(1) Houston

The Houston 2018 dataset is a part of the 2018 IEEE GRSS Data Fusion Competition. It includes Multispectral-LiDAR point cloud data, hyperspectral data, and very-high-resolution RGB imagery. Hyperspectral data was obtained by the ITRES CASI 1500 spectral imaging instrument on the University of Houston campus in Houston, TX, USA. It covers a spectral range of 380–1050 nm with 48 bands and has a ground sampling distance (GSD) of 1 m. The spatial dimensions are 601 × 2384. After normalization, this data is used in this study.

(2) Pavia Center

The Pavia Center dataset was obtained by the Reflective Optical System Imaging Spectrometer (ROSIS) sensor. It was collected over central Pavia, Italy, in 2001. The dataset covers a wavelength range of 430 to 860 nm and contains 102 spectral bands. The spatial dimensions are 1096 × 1096 and the ground sampling distance is 1.3 m. After removing the low-quality areas and bands with low signal-to-noise ratio from the image, the final image size is 1096 × 715 × 102. After normalization, the image is used as the dataset for this study.

(3) Chikusei

Covering agricultural and urban regions in Chikusei, Ibaraki, Japan, the Chikusei dataset was captured using the Headwall Hyperspec-VNIR-C imaging sensor. The ground sampling distance of this dataset is 2.5 m. It consists of 128 spectral bands, and the spectral range covers 363 to 1018 nm. The original spatial dimensions are 2517 × 2335. After removing invalid edge areas, the final size of the Chikusei data is 2304 × 2048 × 128. After normalization, the image is used as the dataset for this study.

### 4.2. Evaluation Metrics and Training Details

We compare EDLGFS against eight advanced methods: the classical bicubic interpolation, 3D-FCNN [24], MCNet [36], LN-atten-CNN [34], G-RDN [46], MSDformer [41], SNLSR [38], and CST [43]. All hyperparameters are kept consistent with their original references as much as possible. However, some parameters were adjusted due to hardware constraints and dataset variations. For example, we have verified that PSNR saturates before 100 epochs for all methods. Therefore, we have uniformly set the epochs to 100. Specifically, due to the high computational cost of MCNet, its batch size was set to 8 for the Chikusei dataset. The parameter settings for the comparison methods are shown in Table 1. Three widely adopted metrics are employed for evaluation: Peak Signal-to-Noise Ratio (PSNR), Structural Similarity (SSIM) and Spectral Angle Mapper (SAM). Their ideal values are +∞ for PSNR, 1 for SSIM, and 0 for SAM.

In the proposed EDLGFS, we employ 3 × 3 kernels for standard convolutions and 1 × 1 kernels for pointwise convolutions. The input channels of the first 3 × 3 convolution correspond to number of bands in the input HSIs. We set the number of feature channels to 96. The numbers of LGFSS and LGFSL are both set to 4. In the LGFS module, the large and small kernel sizes are set to 5 × 5 and 3 × 3, respectively. We use a progressive upsampling strategy [54] to upscale LR HSIs (for example, when the scaling factor is 4, upsampling twice, and when the scaling factor is 8, upsampling three times). The learnable weights $\lambda_{1}$ and $\lambda_{2}$ in the loss function are initialized to 0.95 and 0.05, respectively. The network is trained for 100 epochs using the Adam optimizer with a learning rate of 10^−4^. The batch size is set to 32. All experiments were implemented in PyTorch 2.1.0 on the platform of the National Supercomputing Center in Zhengzhou.

### 4.3. Results of Houston Dataset

For the Houston 2018 dataset, we extract four non-overlapping 256 × 256 × 48 patches from the left region for testing. The remaining region serves as the training set. We augment this data by rotating the images by 90°, 180°, and 270°. Then, we crop the augmented images into 64 × 64 × 48 patches with a spatial overlap of 44 pixels. These patches serve as the reference HR HSIs. According to the Wald protocol [55,56], we generate corresponding LR image patches through band-wise Gaussian filtering. This process constructs the HR and LR image pairs. Specifically, we apply downsampling factors of 2, 4, and 8. This yields LR patches with spatial dimensions of 32 × 32, 16 × 16, and 8 × 8 pixels, respectively.

Table 2 presents the average metrics of all comparative algorithms on the Houston dataset. The best results are highlighted in bold, and the second-best are underlined. For scale factors ×2, ×4, and ×8, all deep learning-based methods consistently outperform the traditional bicubic interpolation method by a significant margin. The proposed EDLGFS achieves the best performance across all scale factors. In terms of PSNR and SSIM, EDLGFS significantly surpasses both traditional and advanced methods. This indicates that the proposed EDLGFS restores the spatial details and structural information more accurately. Additionally, EDLGFS achieves lower SAM values than competing methods. This suggests effective mitigation of spectral distortion and a better balance between spatial enhancement and spectral preservation. EDLGFS demonstrates greater robustness across increasing scale factors (from ×2 to ×8), exhibiting less performance degradation than other methods. Especially in the challenging scale factor ×8, it still maintains a significant advantage, demonstrating its adaptability to diverse resolution demands. Furthermore, we calculate the number of parameters and the computational cost (GFLOPs) during the inference process for a test image, as shown in Table 2. The computational cost of the proposed EDLGFS is significantly lower than that of 3D CNN-based methods. The results indicate that EDLGFS achieves a better balance between reconstruction accuracy and computational efficiency.

**Figure 3:**
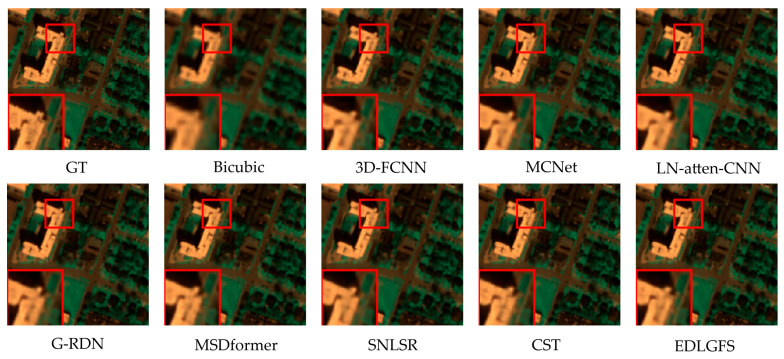
Comparative visual results on the Houston test dataset with spectral bands 16-32-40 as R-G-B (scale ×4), from left to right: Ground Truth (GT), Bicubic, 3D-FCNN, MCNet, LN-atten-CNN, G-RDN, MSDformer, SNLSR, CST, and the proposed EDLGFS.

**Figure 4:**
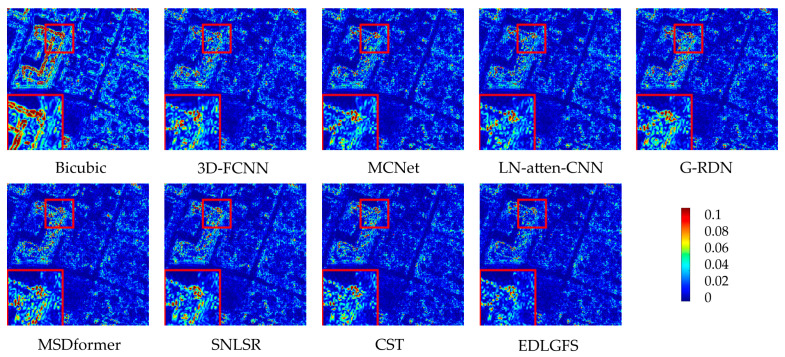
Error maps for the test HSI on Houston (scale ×4).

**Figure 5:**
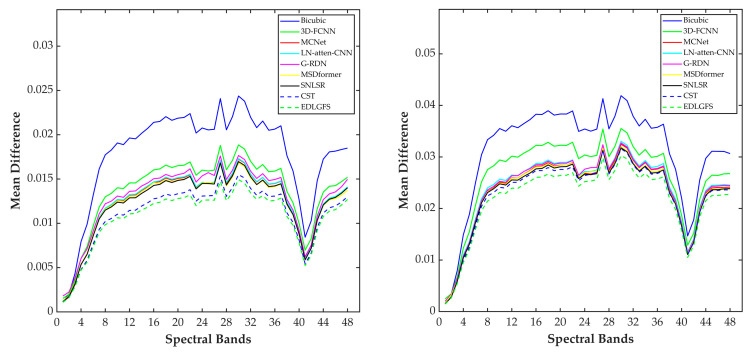
Mean absolute spectral difference curve for the test HSI on Houston (**left**: scale ×4, **right**: scale ×8).

We present qualitative results on the Houston (scale factor ×4) in Figure 3, to visually illustrate the effectiveness of EDLGFS. Pseudo-R-G-B images are generated by combining the 16th, 32nd, and 40th spectral bands. From the results, the image reconstructed by the traditional Bicubic interpolation method is significantly blurred with substantial loss of structural details. Deep learning methods, such as 3D-FCNN, MSDformer, and CST among others, have achieved satisfactory reconstruction quality. However, there is still mild blurring at the building edges, insufficient contour sharpness, and discontinuous local details. In contrast, the proposed EDLGFS produces clearer edges and superior texture details. Through the enlarged picture in the red box, we can see our advantages more clearly. In addition, Figure 4 visualizes the mean error maps across all spectral bands to assess pixel-wise reconstruction accuracy. In the error map, blue and red indicate lower and higher reconstruction errors. As indicated by the enlarged area of the red box, EDLGFS exhibits lower reconstruction errors than other methods, indicating that EDLGFS has superior reconstruction quality. Finally, Figure 5 shows the average spectral difference curves (for scale factors ×4 and ×8) across the test images, which are used to evaluate the spectral reconstruction quality. A lower curve indicates higher spectral consistency with the Ground Truth (GT). These results confirm that EDLGFS achieves the best spectral fidelity across different scales.

### 4.4. Results on Pavia Center Dataset

For the Pavia Center dataset, four test images of size 256 × 256 × 102 are extracted from the left region without overlap. The remaining region is used for training. We first perform data augmentation on this remaining region by rotating it by 90°, 180°, and 270°. Then, we crop the augmented images into 64 × 64 × 102 patches with a spatial overlap of 52 pixels. These patches serve as the reference HR HSIs. We generate corresponding LR image patches from these reference HR image patches through band-wise Gaussian filtering. This process constructs the HR and LR image pairs. Specifically, two, four, and eight Gaussian kernels are used to downsample the HR image patches, yielding their corresponding LR image patches of sizes 32 × 32 × 102, 16 × 16 × 102, and 8 × 8× 102.

For scale factors ×2, ×4, and ×8 on the Pavia Center dataset, Table 3 lists the average performance metrics (PSNR, SSIM, SAM) of all comparison methods. The Pavia Center dataset, acquired in 2001, presents inherent challenges including lower native spatial resolution and a limited available training area. Consequently, the overall results for all methods are lower than those obtained on the Houston dataset. Despite these challenges, the proposed EDLGFS consistently outperforms other methods in terms of PSNR, SSIM, and SAM across all scales. This result further confirms that EDLGFS has stable performance in HSISR. Consistent with the results on the Houston dataset, the computational complexity of EDLGFS on the Pavia Center dataset is significantly lower than that of 3D CNN-based methods. This demonstrates a favorable trade-off between reconstruction accuracy and efficiency.

**Figure 6:**
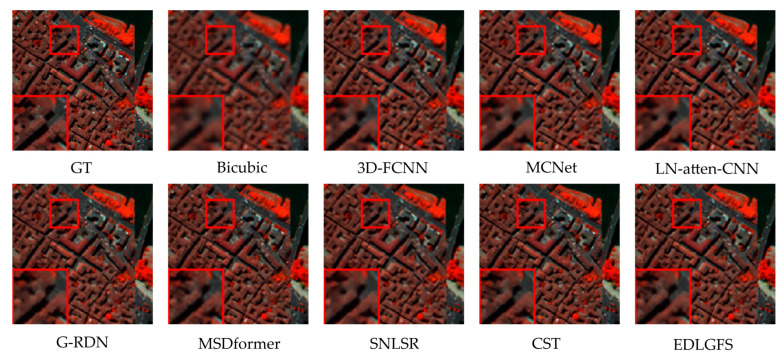
Comparative visual results on the Pavia Center test dataset with spectral bands 96-30-15 as R-G-B (scale ×4), from left to right: Ground Truth (GT), bicubic, 3D-FCNN, MCNet, LN-atten-CNN, G-RDN, MSDformer, SNLSR, CST, and the proposed EDLGFS.

**Figure 7:**
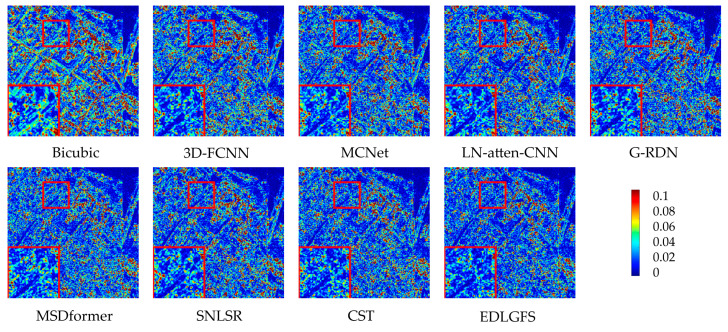
Error maps for the test HSI on Pavia Center (scale ×4).

**Figure 8:**
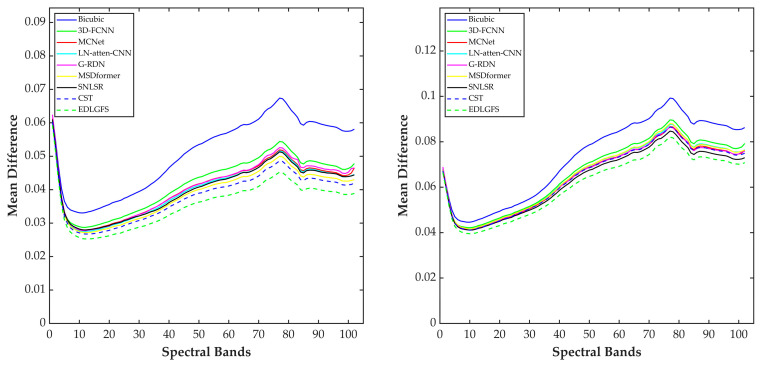
Mean absolute spectral difference curve for the test HSI on Pavia Center (**left**: scale ×4, **right**: scale ×8).

We present the qualitative visualization results of each method on the Pavia Center dataset at a scale factor of ×4 in Figure 6. We select the 96th, 30th, and 15th bands of the images and combine them into pseudo-R-G-B images for visual comparison. Visual inspection reveals that other methods exhibit blurred details. Specifically, building textures and edge contours lack sharpness. However, the proposed EDLGFS demonstrates superior visual fidelity. It achieves overall quality closer to the Ground Truth (GT) and accurately restores edge details and building textures. The enlarged area corresponding to the red box in the figure more intuitively highlights the significant advantage of the proposed EDLGFS in detail restoration. Figure 7 compares error distributions of each method for a scale factor ×4. In Figure 7, the proposed EDLGFS shows a smaller extent of red (high-error) regions, confirming its superior spatial reconstruction accuracy. Finally, the spectral fidelity is evaluated through average spectral difference curves for scale factors ×4 and ×8, as shown in Figure 8. The proposed EDLGFS achieves the lowest spectral difference curves at both scales, demonstrating that it better preserves spectral features.

### 4.5. Results on Chikusei Dataset

For the Chikusei dataset, eight test images of size 256 × 256 × 128 are extracted from the top region without overlap. The remaining region is used for training. We augment this data by rotating the images by 90°, 180°, and 270°. Then, we crop the augmented images into 64 × 64 × 128 patches with a spatial overlap of 24 pixels. These patches serve as the reference HR HSIs. We generate corresponding LR image patches from these reference HR image patches through band-wise Gaussian filtering. This process constructs the HR and LR image pairs. Specifically, two, four, and eight Gaussian kernels are used to downsample the HR image patches, yielding their corresponding LR image patches of sizes 32 × 32 × 128, 16 × 16 × 128, and 8 × 8× 128.

Table 4 lists the quantitative results (PSNR, SSIM, SAM) on the Chikusei dataset for scale factors ×2, ×4, and ×8. The best and second-best values are shown in bold and underlined, respectively. Across all scales, the proposed EDLGFS demonstrates superior performance, leading the comparison both in reconstruction fidelity (PSNR, SSIM) and spectral accuracy (SAM). These results demonstrate that the proposed EDLGFS excels at reconstructing spatial details while preserving spectral quality. This further validates the effectiveness of the edge distillation strategy and the Local–Global Feature Selection mechanism. The proposed EDLGFS has achieved optimal performance across all three test datasets. For the Chikusei dataset, the computational complexity of the proposed EDLGFS is still lower than that of 3D CNN-based methods. These consistent results across diverse datasets demonstrate the strong generalization capabilities of EDLGFS.

**Figure 9:**
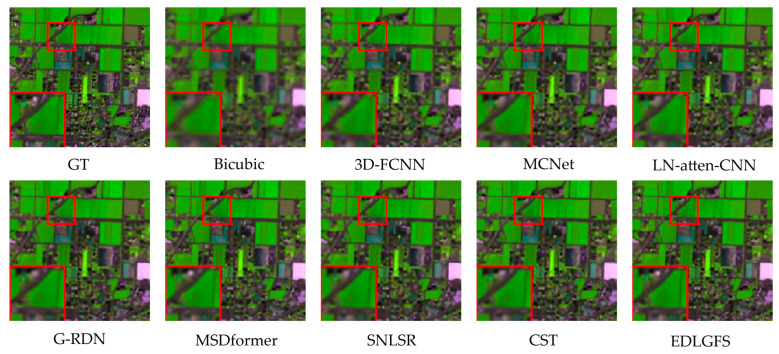
Comparative visual results of the Chikusei test dataset with spectral bands 70-100-36 as R-G-B (scale ×4), from left to right: Ground Truth (GT), bicubic, 3D-FCNN, MCNet, LN-atten-CNN, G-RDN, MSDformer, SNLSR, CST, and the proposed EDLGFS.

**Figure 10:**
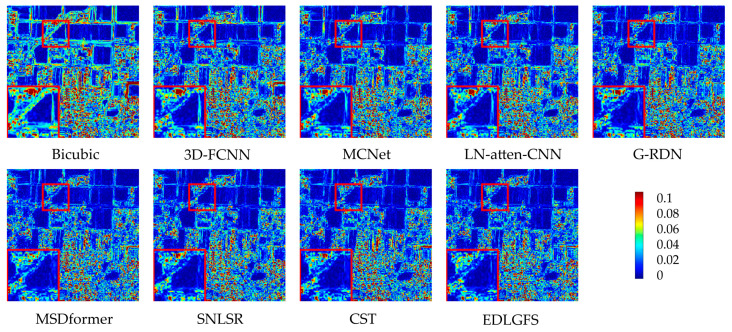
Error maps for the test HSI on Chikusei (scale ×4).

**Figure 11:**
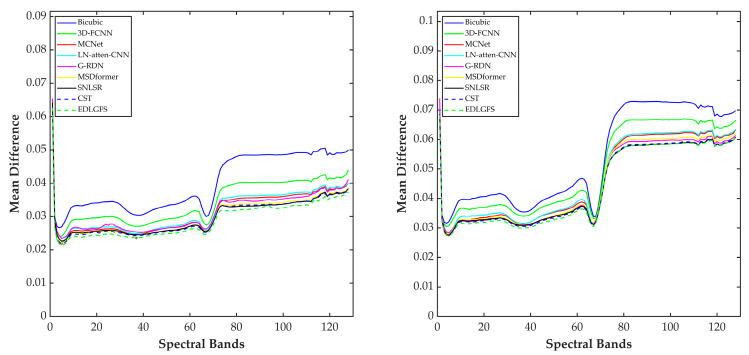
Mean absolute spectral difference curve for the test HSI on Chikusei (**left**: scale ×4, **right**: scale ×8).

Figure 9 presents a qualitative comparison of the Chikusei dataset at a scale factor of 4. For visualization, we select the 70th, 100th, and 36th spectral bands to generate pseudo-R-G-B images. The visualization results reveal that other comparison methods often result in edge blurring. In contrast, the proposed EDLGFS excels at restoring sharp edge details, offering a distinct visual advantage. This can be intuitively verified through the local magnified area marked by the red box. Figure 10 displays the error distribution maps for each method at a scale factor of 4. From the result, EDLGFS exhibits the smallest red (high-error) regions. This indicates minimal deviation from the Ground Truth (GT) and higher reconstruction precision. The average spectral difference curves for scale factors ×4 and ×8 are shown in Figure 11. The proposed EDLGFS achieves the lowest spectral difference curves, demonstrating its ability to effectively enhance spatial resolution while preserving spectral features more accurately than other methods.

### 4.6. Ablation Study

To assess the contribution of each core module to the overall super-resolution performance, an ablation study is conducted in this section. All ablation models are trained and evaluated on the Houston dataset at a scale factor of ×4.

#### 4.6.1. Ablation Study on the Number of LGFSSs

Our core feature reconstruction network consists of a series of LGFSSs. In this section, we investigate the impact of the number of LGFSSs (denoted as N) on the model’s reconstruction ability. As shown in Table 5, when N increases from 3 to 4, the number of model parameters increases from 6.00 M to 7.71 M, and the computational cost (GFLOPs) increases from 34.32 to 41.52. Concurrently, both PSNR and SSIM improve. At this point, PSNR (33.2695) and SSIM (0.9862) reach the optimal level in the table (indicated in bold). As N continues to increase to 5, the number of parameters further rises to 9.43 M, and computational cost increases to 48.71, but metrics such as PSNR and SSIM begin to decline. When N reaches 6, the model parameters quantity reaches 11.14 M, and the computational cost rises to 55.91. Meanwhile, the accuracy of the reconstruction indicators such as PSNR, SSIM, and SAM has significantly decreased. This result indicates that increasing the number of LGFSSs leads to a significant increase in model parameters and computational cost. However, the reconstruction performance of the model does not continuously improve as N increases. We attribute this to overfitting caused by increased depth. Deeper networks typically require larger training datasets to effectively learn feature mappings.

#### 4.6.2. Break-Down Ablation

The proposed EDLGFS integrates three core designs: Edge Distillation, LGFS, and learnable loss weights. We conduct ablation studies on the Houston dataset (×4 and ×8 scaling), Pavia Center dataset (×4 scaling), and Chikusei dataset (×4 scaling) to evaluate the independent contributions of each design element. Each experiment has been independently run five times with five different random seeds. The results are reported as the mean ± standard deviation. Results are summarized in Table 6, Table 7 and Table 8 (best scores in bold). Experiments demonstrate that the proposed EDLGFS exhibits stable performance across all metrics with minimal standard deviation, indicating the model’s good stability. The following analysis is conducted from three aspects: removing the edge distillation branch, removing the LGFS module, and fixing learnable weights.

The design objective of the edge distillation strategy is to guide the model to focus on learning image edge details. When removing the edge branch, the edge-related loss term is disabled, and its corresponding weight is removed. Experiments demonstrate that removing the edge distillation branch leads to performance degradation across all datasets and scaling factors, particularly at higher scaling factors (such as ×8). For example, on the Houston dataset, after removing edge distillation, the PSNR decreases by approximately 0.16 dB and 0.43 dB at ×4 and ×8 scaling factors, respectively. On the Pavia Center and Chikusei datasets, the PSNR decreases by approximately 0.29 dB and 0.18 dB, respectively. This finding demonstrates that the edge distillation strategy effectively enhances the model’s ability to recover edge details. In addition, we only calculate the number of parameters and computational cost (GFLOPs) during the inference process for a test image, as shown in Table 6, Table 7 and Table 8. All parameters and computational costs are calculated using the “thop” library of PyTorch. Notably, the complete model imposes no additional parameters or computational cost during inference compared to the model without the edge branch. This is because the auxiliary edge network is utilized only during training and is discarded during inference. Thus, the strategy improves edge extraction without increasing inference overhead.

LGFS is designed to simultaneously capture both local spatial information and global long-range dependencies within images. The ablation experiment demonstrates that after removing this module, the model’s PSNR on the Houston dataset (×4 scaling) decreases by approximately 0.06 dB, and decreases by approximately 0.07 dB and 0.05 dB on the Pavia Center and Chikusei datasets, respectively. As shown in Table 6, Table 7 and Table 8, the parameters and computational costs significantly decrease after removing LGFS. This indicates that LGFS constitutes the primary component of computational cost in the proposed model. Nevertheless, LGFS significantly enhances the model’s ability to capture local–global features by integrating multi-scale convolution with a self-attention mechanism, thereby bringing stable improvements in multiple evaluation metrics.

The learnable weights in the loss function are designed to dynamically balance the contribution of each loss term. We have tested the impact of fixing the learnable weights to their initial values (0.95, 0.05). The experimental results show that on the Houston dataset (×4 scaling), the model’s PSNR decreases by approximately 0.03 dB. On Pavia Center and Chikusei datasets, the PSNR decreases by approximately 0.03 dB and 0.02 dB, respectively. Although the decrease is small, it still indicates that the learnable weights can adaptively balance the contributions of different loss terms, thereby optimizing the overall performance of the proposed model.

#### 4.6.3. Ablation Study on the Different Convolution Kernel Sizes of LGFS

The proposed LGFS employs convolution with varying kernel sizes to capture features with different receptive fields. This section investigates the impact of different kernel size combinations on model performance. Table 9 presents quantitative comparison results for the Houston test dataset at a scale factor of ×4 (bold indicates optimal metrics). As shown in Table 9, when using the convolution kernel combination of (5 × 5, 3 × 3), the number of parameters size is 7.71 M and the computational cost is 41.52 GFLOPs, which is at a moderate level. Moreover, it simultaneously achieves the optimal level in PSNR, SSIM, and SAM. When using the combination of (3 × 3, 3 × 3) convolution kernels, although the number of parameters and computational cost are the lowest, the model is limited by a fixed receptive field and is difficult to effectively model cross-regional correlations (such as the overall object contours or distant context). For combinations such as (7 × 7, 3 × 3) and (7 × 7, 5 × 5) that involve larger-sized convolution kernels, the number of parameters and computational cost significantly increase. Furthermore, due to overly large receptive fields, the model tends to lose fine-grained local details during training, leading to performance degradation.

#### 4.6.4. Ablation Study on the Different Initial Weights of Loss Function

This section investigates the impact of initial values for learnable weight parameters in the loss function. As shown in Table 10, the model achieves optimal reconstruction results when $\lambda_{1}=0.95$ and $\lambda_{2}=0.05$, achieving optimal values for PSNR, SSIM, and SAM. As $\lambda_{1}$ decreases and $\lambda_{2}$ increases, the accuracy of reconstruction metrics such as PSNR, SSIM, and SAM shows a significant decline. The core cause of this trend is as follows: The loss term corresponding to $\lambda_{1}$ directly restricts the global fitting degree between the model output and the real samples, which is the core constraint to ensure the overall accuracy of the reconstruction result. The loss term corresponding to $\lambda_{2}$ focuses on edge details and belongs to an auxiliary constraint at the detail level. If the proportion of $\lambda_{1}$ decreases and the proportion of $\lambda_{2}$ increases, the model will overly focus on the matching of edge details, thereby weakening the core constraint on the overall reconstruction accuracy and ultimately leading to a decline in overall performance.

#### 4.6.5. Robustness Analysis Against Degradations

Hyperspectral images are often affected by various degradations during imaging, such as noise, transmission errors, or sensor failures. Therefore, evaluating the robustness of super-resolution models against such data variations is crucial. In this section, we explore the reconstruction stability of the proposed EDLGFS under degraded conditions through robustness ablation experiments. To simulate noise effects and sensor failures during imaging, we introduced Gaussian noise and random value degradation into low-resolution image patches during data preparation. As shown in Table 11, the EDLGFS model demonstrates robust stability against both Gaussian noise and random value degradation. The slight degradation in performance is within an acceptable range, demonstrating the model’s adaptability and robustness to common data defects in real-world complex scenarios.

## 5. Conclusions

In this paper, a novel method named EDLGFS is proposed for HSISR. The proposed EDLGFS employs two parallel network branches. The main network learns the complex local–global features and the auxiliary edge network focuses on extracting and refining edge details. These two branches are connected through a knowledge distillation framework, where an edge loss function guides the main network to learn the edge details by the auxiliary edge network. Subsequently, we design a Local–Global Feature Selection mechanism (LGFS). This module first extracts feature representations with varying receptive fields through convolutional kernels of different sizes. Then, it employs the self-attention mechanism to model spatial dependencies between these features. By leveraging these spatial dependencies, it achieves an efficient feature selection mechanism that significantly enhances the ability to capture local–global feature. Additionally, we design a learnable dynamic loss mechanism, which assigns learnable weights to different loss terms, allowing the model to more effectively balance their contributions. Extensive experiments across multiple public datasets demonstrate that the proposed EDLGFS achieves superior reconstruction quality in HSISR.

Although the proposed EDLGFS demonstrates good performance in HSISR, it still has certain limitations. Firstly, the edge distillation branch relies on the Sobel operator for initial edge extraction. This method performs well in most cases, but it is sensitive to noise and complex textures, which may affect the stability of edge guidance in complex scenes. Future work could explore more robust edge detection algorithms or learnable edge extraction modules. Secondly, while the current loss function exhibits adaptability, it primarily optimizes pixel-level errors without sufficiently incorporating perceptual quality or spectral consistency constraints. Developing a more advanced loss function is expected to provide more effective guidance for edge restoration and spectral preservation. Importantly, these limitations do not undermine the validity of our core contributions but rather offer specific directions for future improvements.

## Figures and Tables

**Table 1:** Parameter settings for comparison methods.

Method	Batch Size	Epoch	Learning Rate	Optimizer
3D-FCNN [24]	16	100	0.00005	Adam
MCNet [36]	16/8	100	0.0001	Adam
LN-atten-CNN [34]	16	100	0.001	Adam
G-RDN [46]	16	100	0.0001	Adam
MSDformer [41]	32	100	0.00005	Adam
SNLSR [38]	8	100	0.0002	Adam
CST [43]	32	100	0.0001	Adam
**EDLGFS**	**32**	**100**	**0.** **0001**	**Adam**

**Table 2:** Performance comparison on the Houston test dataset at various scales.

Method	Scale	Param (M)	GFLOPs	PSNR	SSIM	SAM
Bicubic	×2	-	-	34.9599	0.9905	1.6953
3D-FCNN [24]		0.039	123.62	37.8865	0.9954	1.3927
MCNet [36]		1.93	1978.18	38.9117	0.9964	1.2032
LN-atten-CNN [34]		9.78	6396.71	38.8662	0.9963	1.2117
G-RDN [46]		2.17	41.82	38.4582	0.9960	1.3278
MSDformer [41]		10.69	273.80	39.3512	0.9967	1.1631
SNLSR [38]		1.33	38.05	38.8741	0.9964	1.2509
CST [43]		2.83	50.15	39.4362	0.9968	1.1672
**EDLGFS**		7.38	137.68	**39.5342**	**0.9969**	**1.1346**
Bicubic	×4	-	-	29.1727	0.9618	3.2352
3D-FCNN [24]		0.039	123.62	31.1875	0.9776	2.7072
MCNet [36]		2.17	1735.57	31.9065	0.9808	2.5406
LN-atten-CNN [34]		9.90	2219.69	31.9407	0.9811	2.5139
G-RDN [46]		2.17	16.61	31.6579	0.9804	2.5848
MSDformer [41]		12.77	112.62	32.2263	0.9826	2.2550
SNLSR [38]		1.48	12.85	32.0647	0.9817	2.3682
CST [43]		3.16	22.05	33.0054	0.9854	2.1366
**EDLGFS**		7.71	41.52	**33.2695**	**0.9862**	**2.1222**
Bicubic	×8	-	-	24.7235	0.8848	5.5512
3D-FCNN [24]		0.039	123.62	25.7176	0.9172	4.9909
MCNet [36]		2.96	3955.55	26.5633	0.9310	4.6756
LN-atten-CNN [34]		10.29	2315.75	26.4459	0.9295	4.6940
G-RDN [46]		2.17	10.31	26.4514	0.9314	4.4292
MSDformer [41]		14.84	72.32	26.6762	0.9338	4.2053
SNLSR [38]		1.62	10.61	26.6095	0.9332	4.3372
CST [43]		3.49	15.03	26.7080	0.9338	4.1420
**EDLGFS**		8.04	19.74	**27.1169**	**0.9400**	**4.0347**

Bold represents the best. Underlined represents the second-best.

**Table 3:** Performance comparison on the Pavia Center test dataset at various scales.

Method	Scale	Param (M)	GFLOPs	PSNR	SSIM	SAM
Bicubic	×2	-	-	31.1088	0.9393	5.5004
3D-FCNN [24]		0.039	262.70	33.9764	0.9697	4.7763
MCNet [36]		1.93	4203.64	34.9783	0.9754	4.4994
LN-atten-CNN [34]		9.78	13,593.02	34.8647	0.9750	4.4984
G-RDN [46]		2.33	52.10	35.1105	0.9759	4.4454
MSDformer [41]		11.81	427.70	35.2717	0.9767	4.3512
SNLSR [38]		1.45	40.10	35.0469	0.9749	4.4706
CST [43]		2.97	57.02	35.8113	0.9790	4.2460
**EDLGFS**		7.52	144.56	**35.8670**	**0.9792**	**4.2357**
Bicubic	×4	-	-	26.9982	0.8308	7.4899
3D-FCNN [24]		0.039	262.70	28.3387	0.8902	6.8244
MCNet [36]		2.17	3688.09	28.5679	0.8964	6.7685
LN-atten-CNN [34]		9.90	4716.85	28.6142	0.8975	6.7592
G-RDN [46]		2.33	26.51	28.4978	0.8956	6.7869
MSDformer [41]		13.89	162.56	28.8341	0.9029	6.5861
SNLSR [38]		1.59	13.44	28.5555	0.8940	6.6835
CST [43]		3.30	28.36	28.9894	0.9075	6.4145
**EDLGFS**		7.85	47.82	**29.4384**	**0.9149**	**6.2443**
Bicubic	×8	-	-	24.3234	0.6416	9.3097
3D-FCNN [24]		0.039	262.70	24.9446	0.7297	8.8270
MCNet [36]		2.96	8405.54	25.1314	0.7473	8.7901
LN-atten-CNN [34]		10.29	4920.97	25.0883	0.7446	8.8178
G-RDN [46]		2.33	20.12	25.1309	0.7488	8.7251
MSDformer [41]		15.96	96.27	25.1389	0.7435	8.6188
SNLSR [38]		1.74	10.83	25.1438	0.7473	8.6372
CST [43]		3.63	21.19	25.1965	0.7518	8.5790
**EDLGFS**		8.18	25.90	**25.5081**	**0.7687**	**8.2387**

Bold represents the best. Underlined represents the second-best.

**Table 4:** Performance comparison of the Chikusei test dataset at various scales.

Method	Scale	Param (M)	GFLOPs	PSNR	SSIM	SAM
Bicubic	×2	-	-	34.2497	0.9693	2.6459
3D-FCNN [24]		0.039	329.66	37.5895	0.9858	2.0777
MCNet [36]		1.93	5275.16	38.9705	0.9893	1.8702
LN-atten-CNN [34]		9.78	17,057.90	38.9609	0.9893	1.8770
G-RDN [46]		2.43	58.28	39.1192	0.9895	1.8678
MSDformer [41]		12.31	494.48	39.6503	0.9905	1.7618
SNLSR [38]		1.50	41.08	39.3613	0.9892	1.8795
CST [43]		3.04	60.34	39.7611	0.9907	1.7507
**EDLGFS**		7.59	147.88	**39.8614**	**0.9909**	**1.7243**
Bicubic	×4	-	-	29.2219	0.8975	4.5112
3D-FCNN [24]		0.039	329.66	30.5396	0.9303	3.9475
MCNet [36]		2.17	4628.20	31.5212	0.9445	3.5525
LN-atten-CNN [34]		9.90	5919.18	31.4605	0.9437	3.5722
G-RDN [46]		2.43	32.51	31.5824	0.9451	3.5622
MSDformer [41]		14.38	184.77	31.9485	0.9496	3.2910
SNLSR [38]		1.65	13.72	31.7918	0.9470	3.3576
CST [43]		3.37	31.39	32.0329	0.9506	3.2436
**EDLGFS**		7.92	50.86	**32.1864**	**0.9524**	**3.2159**
Bicubic	×8	-	-	26.3401	0.7845	6.4412
3D-FCNN [24]		0.039	329.66	26.9218	0.8278	5.9352
MCNet [36]		2.96	10,548.13	27.3872	0.8466	5.5592
LN-atten-CNN [34]		10.29	6175.33	27.3448	0.8456	5.6082
G-RDN [46]		2.43	26.06	27.3936	0.8474	5.5645
MSDformer [41]		16.46	107.35	27.4727	0.8511	5.3929
SNLSR [38]		1.80	10.94	27.6038	0.8542	5.3245
CST [43]		3.70	24.16	27.6039	0.8548	5.2503
**EDLGFS**		8.25	28.87	**27.6864**	**0.8595**	**5.1611**

Bold represents the best. Underlined represents the second-best.

**Table 5:** Quantitative evaluation of the number of LGFSSs in the Houston test dataset (scale ×4).

Number (N)	Param (M)	GFLOPs	PSNR	SSIM	SAM
3	6.00	34.32	33.2323	0.9861	2.1258
4	7.71	41.52	**33.2695**	**0.9862**	**2.1222**
5	9.43	48.71	33.2289	0.9860	2.1457
6	11.14	55.91	33.0614	0.9855	2.1830

Bold represents the best.

**Table 6:** Break-down ablation study. We report the testing results on the Houston test dataset (scale ×4 and scale ×8).

Edge Distilled	LGFS	Learnable Weights	Scale	Param (M)	GFLOPs	PSNR	SSIM	SAM
√	√	√	×4	7.71	41.52	**33.** **2471 ± 0.0219**	**0.986** **1 ± 0.0001**	2.1266 ± 0.0075
×	√	×		7.71	41.52	33.0910 ± 0.0285	0.9857 ± 0.0001	**2.** **1189 ± 0.0083**
√	×	√		2.55	19.57	33.1917 ± 0.0330	0.9859 ± 0.0001	2.1341 ± 0.0019
√	√	×		7.71	41.52	33.2129 ± 0.0231	0.9860 ± 0.0001	2.1455 ± 0.0099
√	√	√	×8	8.04	19.74	**27.1146** ** ± 0.0237**	**0.9398** ** ± 0.0002**	**4.0776** ** ± 0.0350**
×	√	×		8.04	19.74	26.6879 ± 0.0276	0.9339 ± 0.0005	4.1574 ± 0.0155
√	×	√		2.88	14.41	26.8651 ± 0.1018	0.9361 ± 0.0016	**4.0776** ** ± 0.0210**
√	√	×		8.04	19.74	27.0779 ± 0.0297	0.9395 ± 0.0003	4.0815 ± 0.0159

Bold represents the best. √ indicates that this module is present; × indicates that this module has been excluded.

**Table 7:** Break-down ablation study. We report the testing results on the Pavia Center test dataset (scale ×4).

Edge Distilled	LGFS	Learnable Weights	Scale	Param (M)	GFLOPs	PSNR	SSIM	SAM
√	√	√	×4	7.85	47.82	**29.4675 ± 0.0178**	**0.9156 ± 0.0004**	**6.2211 ± 0.0133**
×	√	×		7.85	47.82	29.1791 ± 0.0360	0.9106 ± 0.0007	6.2816 ± 0.0174
√	×	√		2.69	25.88	29.4004 ± 0.0271	0.9141 ± 0.0005	6.2862 ± 0.0184
√	√	×		7.85	47.82	29.4406 ± 0.0325	0.9151 ± 0.0008	6.2571 ± 0.0355

Bold represents the best. √ indicates that this module is present; × indicates that this module has been excluded.

**Table 8:** Break-down ablation study. We report the testing results on the Chikusei test dataset (scale ×4).

Edge Distilled	LGFS	Learnable Weights	Scale	Param (M)	GFLOPs	PSNR	SSIM	SAM
√	√	√	×4	7.92	50.86	**32.1509 ± 0.0333**	**0.9519 ± 0.0004**	**3.2037 ± 0.0118**
×	√	×		7.92	50.86	31.9663 ± 0.0360	0.9501 ± 0.0003	3.2488 ± 0.0106
√	×	√		2.75	28.92	32.1039 ± 0.0342	0.9514 ± 0.0003	3.2181 ± 0.0132
√	√	×		7.92	50.86	32.1283 ± 0.0314	0.9517 ± 0.0004	3.2071 ± 0.0087

Bold represents the best. √ indicates that this module is present; × indicates that this module has been excluded.

**Table 9:** Quantitative evaluation of the different convolution kernel sizes of LGFS on the Houston test dataset (scale ×4).

Kernel Size	Param (M)	GFLOPs	PSNR	SSIM	SAM
(3 × 3, 3 × 3)	5.36	31.85	33.1820	0.9859	2.1543
(5 × 5, 3 × 3)	7.71	41.52	**33.2695**	**0.9862**	**2.1222**
(7 × 7, 3 × 3)	11.25	56.01	33.1853	0.9859	2.1246
(7 × 7, 5 × 5)	13.61	65.68	33.1045	0.9857	2.1464

Bold represents the best.

**Table 10:** Quantitative evaluation of the different initial weights of loss function on the Houston test dataset (scale ×4).

$(\lambda_{1}$ $,\;\lambda_{2})$	PSNR	SSIM	SAM
(0.95, 0.05)	**33.2695**	**0.9862**	**2.1222**
(0.9, 0.1)	33.1545	0.9858	2.1666
(0.85, 0.15)	32.9368	0.9850	2.2003
(0.8, 0.2)	32.7982	0.9845	2.2214

Bold represents the best.

**Table 11:** Comparison of the SR reconstruction performance of the proposed EDLGFS under different degradation conditions.

Degradation Type	PSNR	SSIM	SAM
Raw Data	**33.2695**	**0.9862**	**2.1222**
Noisy Data	32.9522	0.9852	2.1688
Random Degradation	32.7938	0.9849	2.2020

Bold represents the best.

## Data Availability

The data presented in this study are available on request from the authors.
